# Analysis of measurement changes in pelvic incidence according to pelvic rotation using a three-dimensional model

**DOI:** 10.1186/s12891-022-05063-9

**Published:** 2022-02-02

**Authors:** Ki Young Lee, Jung-Hee Lee, Sang-Kyu Im, Won Young Lee

**Affiliations:** Department of Orthopaedic Surgery, Graduate School, College of Medicine, Kyung Hee University, 23, Kyungheedae-ro, Dongdaemun-gu, Seoul, 130-872 South Korea

**Keywords:** Pelvis, Rotation, Pelvic incidence

## Abstract

**Background:**

Pelvic incidence (PI) is used as a key parameter in surgical correction of adult spinal deformity (ASD). However, reflecting the exact center or inclination of the three-dimensional anatomical structures on the two-dimensional (2D) sagittal radiographs is limited, resulting in measurement errors. Therefore, we evaluated whether there is a change in PI measurement according to the actual rotation of the pelvis, and conducted a study on a more accurate method for PI measurement using 2D sagittal radiographs.

**Methods:**

From 2014 to 2015, the data of 30 patients who visited our outpatient clinic were analyzed retrospectively. CT scans including those of the lower lumbar spine, pelvis, and both femurs in the DICOM format were imported to Mimics Research 17.0 (Materialise NV, Belgium), SolidWorks (Dassault systems, France), and AutoCAD 2014 (AUTODESK, US). The changes in PI according to vertical and horizontal pelvic rotations were evaluated.

**Results:**

The average PIs according to the horizontal pelvic rotations measured on AutoCAD with 0°, 5°, 10°, 15°, 20°, 25°, 30°, 35°, and 40° were 48.8°, 48.7°, 48.3°, 47.8°, 46.9°, 45.6°, 44.0°, 42.2°, and 39.9°, respectively. The PI with an acceptable error of 6° on radiographs was 35° in the horizontal pelvic rotation. The average PIs according to the vertical pelvic rotations measured on AutoCAD with 0°, 5°, 10°, 15°, 20°, 25°, 30°, 35°, and 40° were 48.8°, 49.0°, 49.5°, 50.2°, 51.3°, 52.7°, 54.4°, 56.6°, and 59.4°, respectively. The PI with an acceptable error of 6° on radiographs was 30° in the vertical pelvic rotation.

**Conclusions:**

This study revealed that the PI value could differ from the actual anatomical value due to the horizontal and vertical rotation of the pelvis while acquiring the radiograph. Regarding whole-spine lateral radiographs, errors in PI measurement may occur due to pelvic rotation or nonvertical projection of X-rays. In the standing pelvic lateral radiographs, ensuring superposition of the femoral heads at the center and obtaining the straight sacral endplate by referring to CT or magnetic resonance imaging would be a more accurate measurement method to define PI.

## Background

Optimal sagittal balance is an important factor in maintaining an efficient and stable posture, absorbing the load on the spine effectively, and maximizing the performance of the paraspinal muscles [1]. The sagittal balance of the spine is influenced by the spinal curvature, including thoracic kyphosis (TK) and lumbar lordosis (LL), and the position and angle of the spine, pelvis, hip joint, and knee joint. Notably, as the key component of the overall sagittal balance is the compensation cascade in the pelvis, understanding of the relationship between the pelvis and spine is essential [1]. The importance of restoring the optimal sagittal balance in patients with adult spinal deformity (ASD) has been also well recognized [2–4]. In the surgical treatment of patients with ASD, the restoration of the optimal sagittal balance requires preoperative radiological measurement of the sagittal curvature and dynamic factors such as the correlation between the pelvis and hip joint [1].

The spine and pelvis are closely related and show a chain of correlation [5]. The pelvis is the foundation of the spine, and the importance of evaluating the spinopelvic balance based on pelvic morphology has been highlighted in previous studies [6]. In ASD surgery, the assessment of the pelvic parameters that define the sagittal pelvic alignment can be broadly divided into two categories: an anatomic parameter, the pelvic incidence (PI), and two positional parameters, the pelvic tilt (PT) and sacral slope (SS) [7]. PT and SS are the parameters determined by the vertical or horizontal reference line associated with the position or orientation of the pelvis. PI was first described by Duval-Baupère et al. [7], and it is the most commonly used anatomical parameter of the pelvis. It is defined as the angle between the perpendicular line from the sacral plate and the line connecting the midpoint of the sacral plate to the midpoint of the bicoxofemoral axis [7]. Legaye et al. [8] stated that PI is the fundamental pelvic parameter for three-dimensional (3D) regulation of sagittal spinal curves. Additionally, because it is a stably maintained anatomical parameter, even in an arbitrary position and orientation [9], it serves as the key parameter in the surgical treatment of patients with ASD [10].

Nevertheless, the pelvic parameters measured in most previous studies have been assessed using two-dimensional (2D) sagittal radiographs in the standing position [8], limiting the reflection of the accurate center or inclination in 3D anatomical structures [11]. In particular, due to the rotation of the pelvis or nonvertical projection of the X-ray in whole-spine lateral radiography, it is difficult to obtain the superposition of two femoral heads in practice [12]. This results in errors in PI measurements as they are taken based on the midpoint on the line connecting the centers of the femoral heads as the reference point of the hip axis [12]. The accuracy of radiologic measurements such as the spinopelvic parameters, especially PI, is crucial in the preoperative or postoperative evaluation of patients with ASD. Therefore, in this study, the CT scans in the DICOM format of the pelvic CT for the treatment and diagnosis of patients with ASD were analyzed using Mimics Research 17.0 × 64 (Materialise NV, Belgium), SolidWorks (Dassault Systems, France), and AutoCAD 2014 (AUTODESK, US). This study aimed to evaluate PI changes according to the actual pelvic rotation and determination of the more accurate method for PI measurement using 2D sagittal radiographs.

## Methods

### Patient selection

The subjects in this study were 84 patients who visited the outpatient clinic at the study hospital between February 2014 and March 2015 for surgical or nonsurgical treatment.

The inclusion criteria were as follows: (i) patients aged ≥20 years and (ii) patients with radiographs and 3D CT scans of the spine and pelvis for diagnosis and treatment. The exclusion criteria were as follows: patients with a deformity resulting from coxofemoral pathology, neuromuscular spinal deformity, spinal infection, inflammatory disease such as ankylosing spondylitis, and tumorous condition.

### PI measurement according to pelvic rotation (Fig. 1)

We imported CT scans including those of the lower lumbar spine, pelvis, and both femurs in the DICOM format to Mimics Research 17.0 × 64 (Materialise NV, Belgium). We performed and modified 3D reconstruction images using the 3-Matic program. In brief, the program was used to segment the left and right femoral heads into halves based on the coronal, sagittal, and axial planes (Fig. 2). After segmentation along the coronal and sagittal planes on the axial plane of the S1 endplate, the left sacrum and lower lumbar areas were deleted (Fig. 3). The resulting 3D reconstruction model was applied in SolidWorks (Dassault Systems, France) to produce 3D CAD drawings and for distance measurement. First, the three-point method was used for drawing circles along the segmented left and right femoral heads (Fig. 4A and B), and a 3D sketch was drawn for the line connecting the center points of the circles (Fig. 4C). Next, a line was drawn along the segmented endplate of the sacrum on the sagittal plane, and the vertical line was drawn at the center point of the line (Fig. 4D). The center of the line connecting the center of the S1 endplate and that of the femoral head was defined using a 3D sketch (Fig. 5A). The planes were tilted to 5° angular intervals (horizontal and vertical rotation of the pelvis: 0°, 5°, 10°, 15°, 20°, 25°, 30°, 35°, and 40°) for the sagittal plane based on the vertical and horizontal reference lines (Fig. 5B and C). These planes positioned at an interval of 5° were used to adjust the view in vertical and horizontal directions on SolidWorks. Each captured screen was imported to AutoCAD 2014 (AUTODESK, US) to measure PI angle (Fig. 6).

**Fig. 1:**
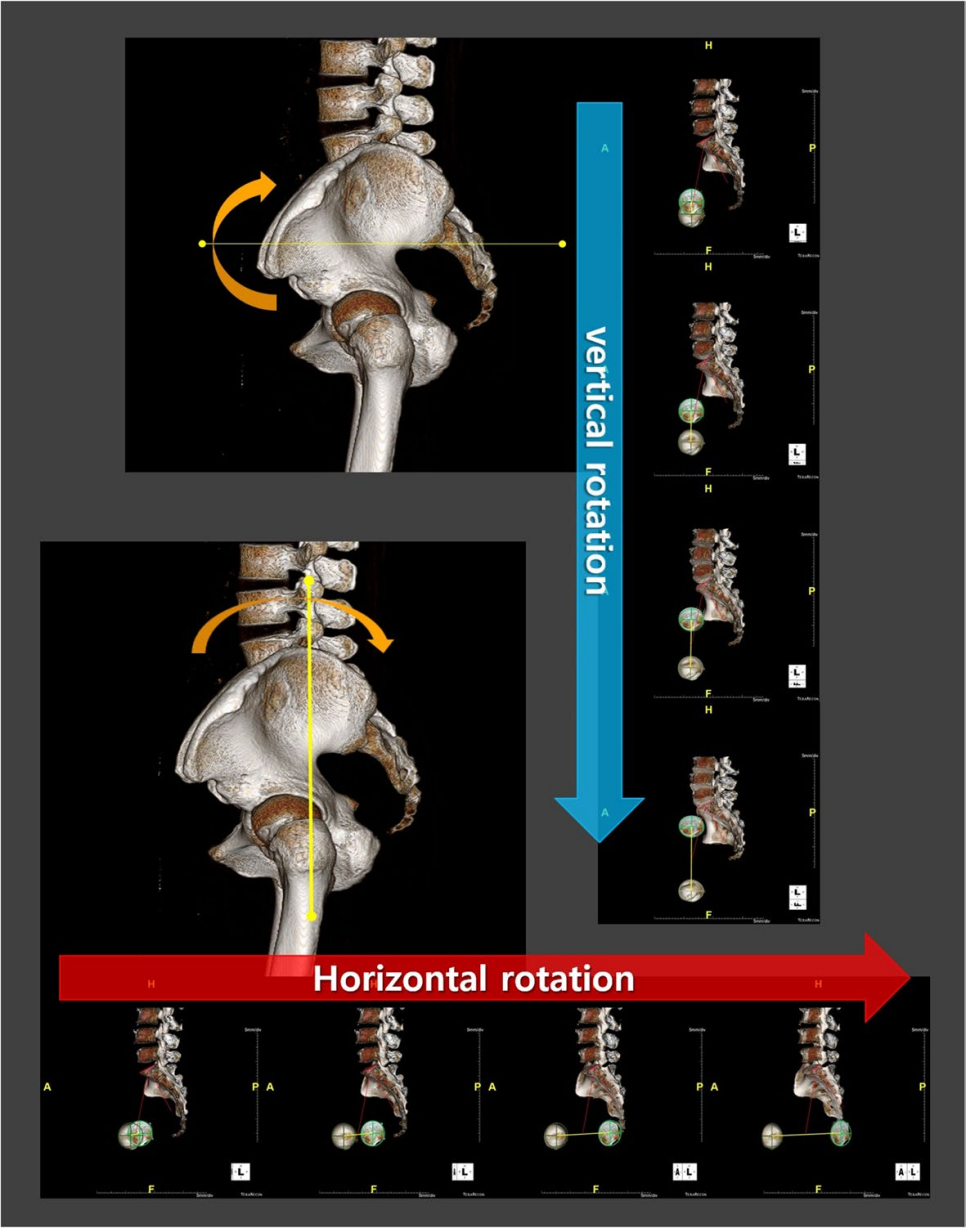
Pelvic incidence evaluation according to the horizontal and vertical pelvic rotation

**Fig. 2:**
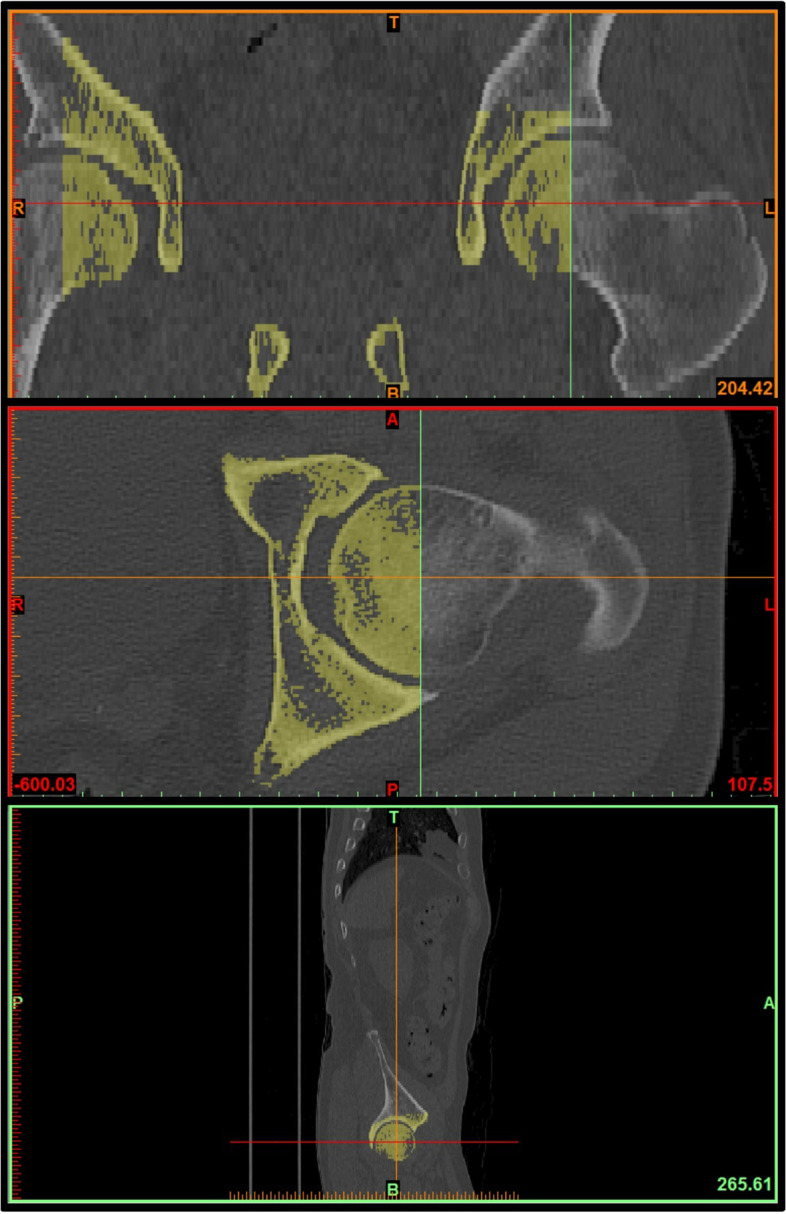
3D reconstruction of femoral head for pelvic incidence; Acquired CT images were performed and modified 3D reconstruction images using 3-Matic program. Segmentation of both femoral heads into halves based on the coronal, sagittal, and axial plane

**Fig. 3:**
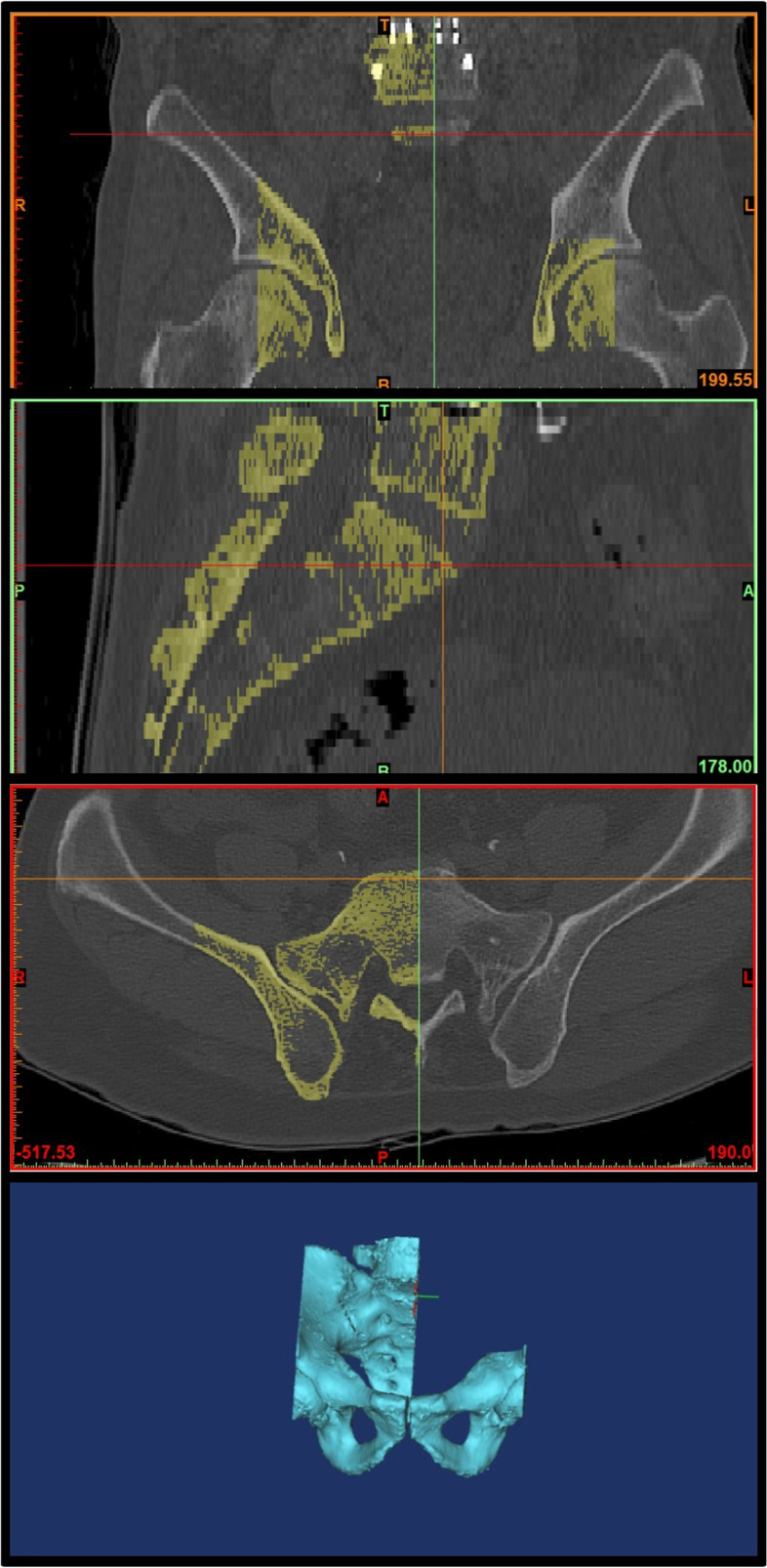
3D reconstruction of sacrum and lower lumbar for pelvic incidence; Identification of S1 end plate of CT scans and segmentation of S1 endplate in coronal, sagittal, and axial plane. Remove left sacrum and lower lumbar images

**Fig. 4:**
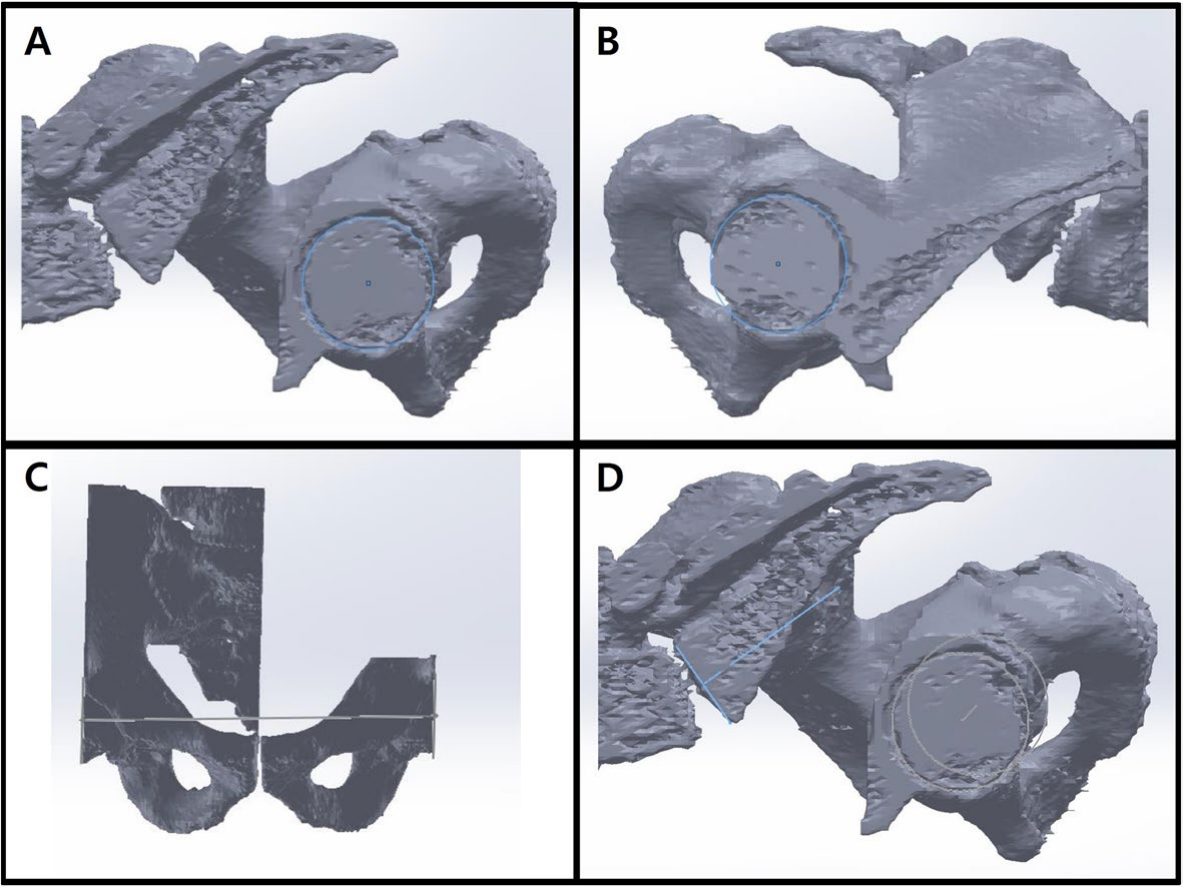
3D sketch of both femoral heads and S1 end plate; The completed 3D reconstruction model was applied in SolidWorks (Dassault systems, France) software to produce 3D sketch ​​of the 3D model. **A** and **B** Circle drawings along the segmented left and right femoral heads. **C** Drawing line connecting the center points of the circles. **D** Drawing line along the segmented endplate of the sacrum on the sagittal plane and vertical line at the center point of the line

**Fig. 5:**
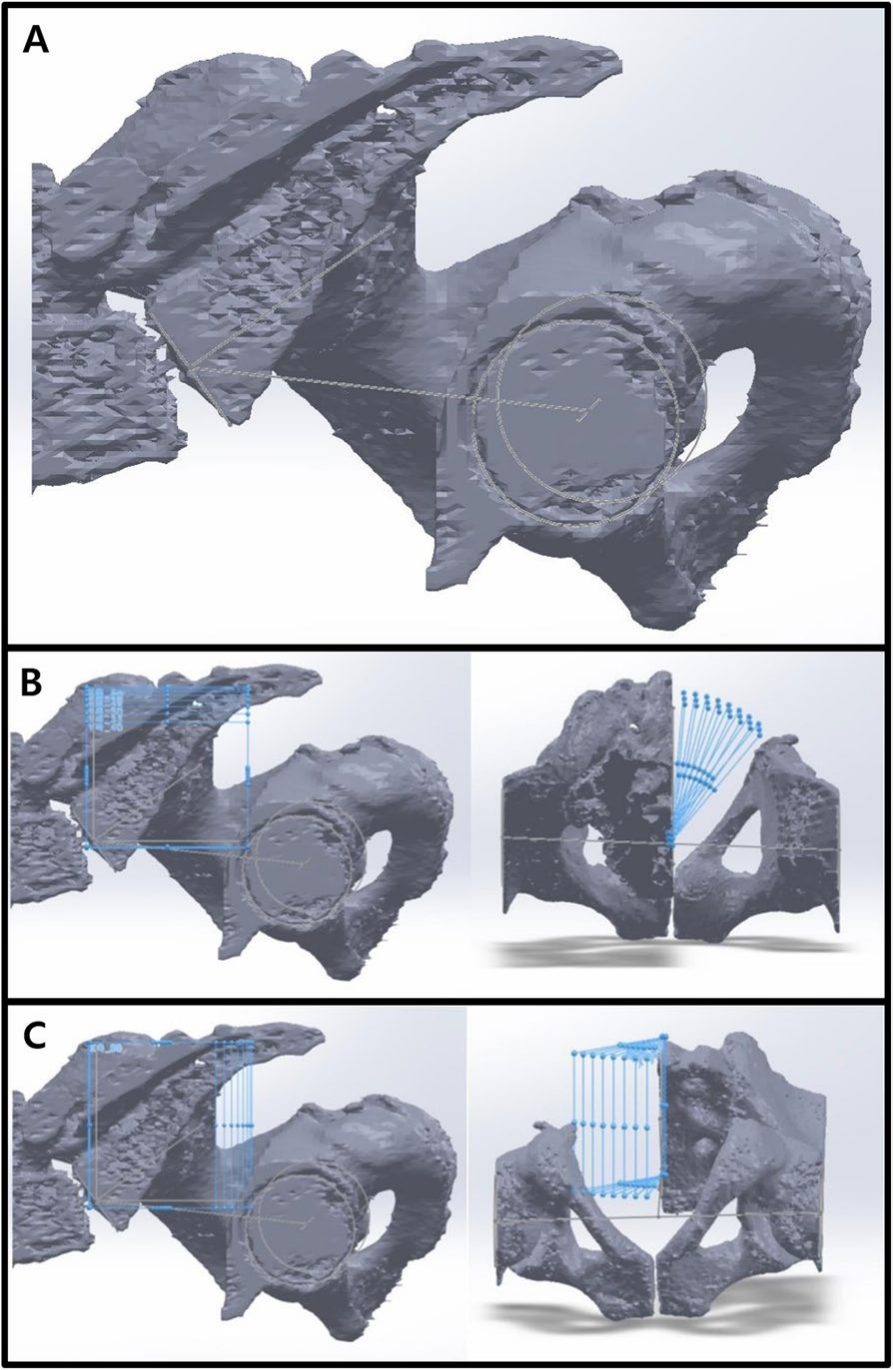
Horizontal and vertical rotation of the sagittal plane with a 3D sketch; **A** The center point of the line connecting the center of the S1 endplate and the center of the femoral head was defined. **B** and **C** The planes were tilted to 5° angular intervals (the horizontal and vertical rotation of the pelvis: 0°, 5°, 10°, 15°, 20°, 25°, 30°, 35°, and 40°) for the sagittal plane based on the vertical and horizontal reference lines

**Fig. 6:**
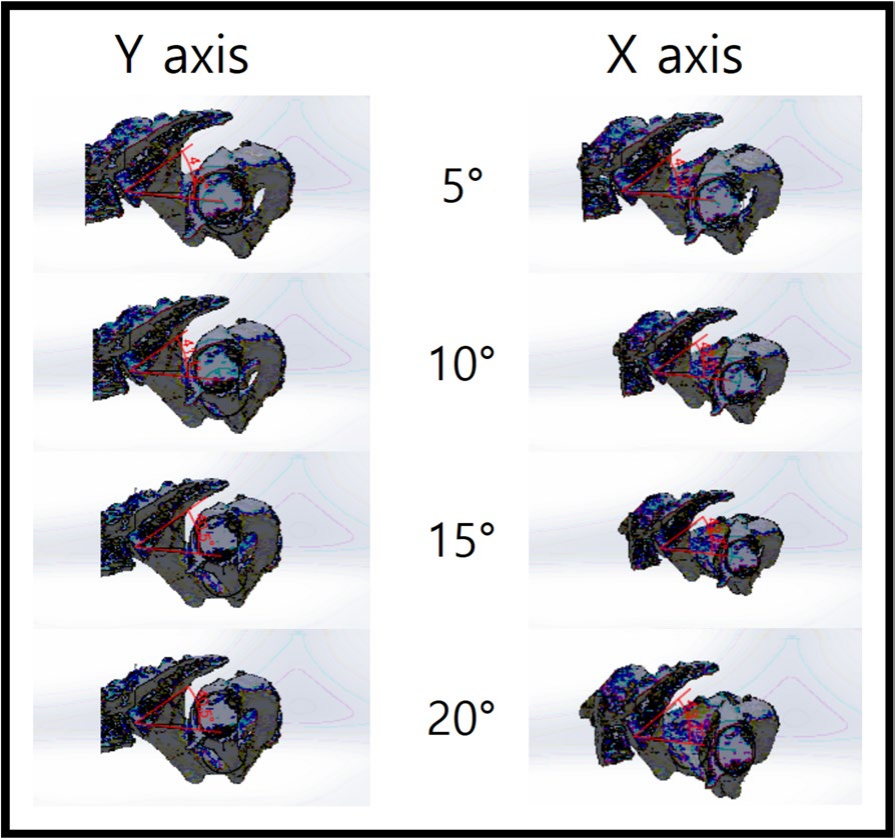
Pelvic incidence measurement in various plane; Planes in the interval of 5° angles were used to adjust the view in Solidworks’ vertical and horizontal directions, and each captured screen was imported to AutoCAD 2014 (AUTODESK, US) to measure the angle of the PI

In a study by Lazennec et al. [13], which was conducted on 81 patients, a mean of 6° variability was reported for satisfactory reproducibility of repeated angle measurements. In this study, the PI of an acceptable error of 6° in AutoCAD measurements according to the horizontal and vertical pelvic rotation was measured.

### Statistical analysis

All statistical analyses were performed using SPSS (version 20.0, SPSS Inc., Chicago, IL, USA). To evaluate the interobserver reliability for the measurement, the analyses and measurements of the 3D models were performed by two of the authors with two engineers who specialized in spinopelvic imaging trained by orthopedic and radiologic professors at our clinics (K.M.L. with K.Y.L., and M.J.J. with S.K.I.). The intraclass correlation coefficients (ICCs) (two-way mixed effects model, ICC model 3.1) were calculated to evaluate the consistency between observers and between measurements of a single observer. The reliability was measured on a scale of 0 to 1, with > 0.75 considered as excellent, 0.40–0.75 as fair to good, and < 0.40 as poor [14].

## Results

### Baseline characteristics of the patients (Table 1)

At the time of the study, the database included 84 patients. After applying the inclusion criteria, 30 patients were identified for analysis. The patients included 11 men and 19 women, and their average age was 50.1 years. Eleven patients were diagnosed with degenerative spondylolisthesis, 8 with spinal stenosis, 6 with degenerative disc disease, and 5 with a compression fracture.

**Table 1:** Demographics and Baseline Data (30 cases) †

Variable	Mean ± SD or number
Gender	
Male	11
Female	19
Age (years)	50.1 ± 0.8
Degenerative spondylosis	11
Spinal stenosis	8
Degenerative disc disease	6
Compression fracture	5

† Data are presented as mean ± standard deviation or number

### PI according to horizontal pelvic rotation (Table 2)

The average PIs according to the horizontal pelvic rotation measured on AutoCAD with 0°, 5°, 10°, 15°, 20°, 25°, 30°, 35°, and 40° were 48.8°, 48.7°, 48.3°, 47.8°, 46.9°, 45.6°, 44.0°, 42.2°, and 39.9°, respectively. Based on the 3D model with 0° horizontal rotation, the percentage (%) changes in each angle were − 0.31, − 1.01, − 2.18, − 4.05, − 6.56, − 9.82, − 13.62, and − 18.20, respectively. The PI of an acceptable error of 6° on radiographs [12, 13] was 35° in the horizontal pelvic rotation.

**Table 2:** Effect of horizontal pelvis rotation on PI (30 cases) †

Horizontal rotation	0°	5°	10°	15°	20°	25°	30°	35°	40°
Pelvic incidence	48.8	48.7	48.3	47.8	46.9	45.6	44.0	42.2	39.9
Change (%)		−0.31	−1.01	−2.18	−4.05	−6.56	−9.82	−13.62	− 18.20

† Data are presented as mean values

The ICCs of PI measurements according to the horizontal pelvic rotation were classified as excellent, with an intraobserver ICC of 0.97 and interobserver ICC of 0.94.

### PI according to vertical pelvic rotation (Table 3)

The average PIs according to the vertical pelvic rotation measured on AutoCAD with 0°, 5°, 10°, 15°, 20°, 25°, 30°, 35°, and 40° were 48.8°, 49.0°, 49.5°, 50.2°, 51.3°, 52.7°, 54.4°, 56.6°, and 59.4°, respectively. Based on the 3D model with 0° vertical rotation, the percentage (%) changes in each angle were + 0.38, + 1.28, + 2.73, + 5.02, + 7.88, + 11.34, + 15.98, and + 21.55. The PI of an acceptable error of 6° on radiographs [12, 13] was 30° in the vertical pelvic rotation.

**Table 3:** Effect of vertical pelvis rotation on PI (30 cases) †

Vertical rotation	0°	5°	10°	15°	20°	25°	30°	35°	40°
Pelvic incidence	48.8	49.0	49.5	50.2	51.3	52.7	54.4	56.6	59.4
Change (%)		+ 0.38	+ 1.28	+ 2.73	+ 5.02	+ 7.88	+ 11.45	+ 15.98	+ 21.55

† Data are presented as mean values

The ICCs of PI measurements according to the vertical pelvic rotation were classified as excellent, with an intraobserver ICC of 0.95 and interobserver ICC of 0.96.

## Discussion

There has been increasing recognition of the “chain of correlations” extending from the pelvic alignment to the spine [5] and that PI is a standard measurement for the surgical treatment of patients with ASD. Various formulas related to PI have been proposed for the surgical treatment of ASD to date [15, 16]. For instance, Schwab et al. [17] suggested a simple formula (LL = PI + 9 [±9]) to estimate the mean lumbar lordosis from the mean PI. Accurate PI measurement is thus a prerequisite for spine surgeons in the treatment of patients with ASD.

### PI and pelvic rotation

PI is generally measured as the angle between the perpendicular line from the sacral plate and the line connecting the midpoint of the sacral plate to the midpoint of the bicoxofemoral axis on 2D sagittal radiographs of standing whole-spine lateral radiographs [7]. However, a 3D image of the pelvis from 2D radiographs can be influenced by pelvic position and orientation [12], for which there is a difficulty in precisely identifying the sacral endplate and bicoxofemoral axis [18]. In addition, radiological measurements, including PI measurement, may be influenced by the surgeon’s knowledge and consequent experience related to the anatomical landmarks [6].

In clinical practice, malposition or malorientation of the pelvis is commonly observed using standing whole-spine lateral radiographs because of factors such as the patient’s incorrect standing position, pelvic obliquity due to leg length discrepancy, and divergent X-ray beam, which could cause an error in the measurement of spinopelvic parameters [19, 20]. Thus, in 1998, Jackson et al. [19] highlighted the need for an accurate imaging technique for the pelvis to achieve more precise radiological measurements, including those of PI. They proposed geometrical rules to show that all radiographs presented 15° or less vertical pelvic rotation with simultaneous 20° or less tilt on the horizontal plane.

Tyrakowski et al. [12], using a single radiological phantom, defined 0° rotation as the complete superposition of the femoral heads in the anteroposterior direction on lateral radiographs and produced radiographs through rotation at 5° intervals up to 45° along the vertical axis. As a result, PI was shown to vary according to the pelvic position on the axial plane. The proper maximal angle of pelvic rotation for a reliable PI measurement on lateral radiographs was reported to be 30°. They also reported 2 years later in a study on PI measurements based on horizontal pelvic rotation that PI may be influenced by pelvic rotation on the coronal plane upon radiography and that a substantial error of PI measurements may occur upon 20° or more horizontal rotation [20]. In our study, similar results were obtained in that PI of an acceptable error of 6° on radiographs [12, 13] was 35° in horizontal pelvic rotation and 30° in vertical pelvic rotation.

This study agrees with the two previous studies by Tyrakowski et al. [12, 20] in that the changes in PI according to the horizontal or vertical rotation of the pelvis were analyzed. However, the key difference lies in the PI measurement method. Although the conventional measurements were based on simple radiological scans, as in the studies by Tyrakowski et al. [12, 20], the measured values cannot be accurately reproduced by repeated measurements with a constant probability of both intra- and inter-rater errors. In the present study, on the contrary, a higher reliability of result values could be achieved using CT scans and conducting 3D measurements using a 3D model based on several specialized programs, including AutoCAD. Another notable difference from the studies by Tyrakowski et al. [12, 20], where a single radiological phantom was used, is that the measurements in this study were taken from 30 patients. Through such highly reliable of patients, we revealed that PI measurements could be influenced by the horizontal and vertical rotation (0°, 5°, 10°, 15°, 20°, 25°, 30°, 35°, and 40°) of the pelvis while acquiring the radiograph.

### Optimal PI evaluation

For an ideal assessment of pelvic parameters, including PI, it is crucial to acquire radiographs that allow precise identification of the sacral endplate in a straight line with two overlapping femoral heads [6]. Despite this, spinopelvic parameters are usually measured on 36-in.-long cassette lateral radiographs of the spine, and the projection of whole-spine radiographs is centered on the 12th vertebra [18]. Therefore, obtaining the perfect superposition of the two femoral heads and precisely identifying the sacral endplate are usually impossible using whole-spine radiographs. In particular, the sacral endplate could show an overlap of the lumbar spine and pelvic bony structures on whole-spine radiographs. At the same time, the presence of a buttock or ilium shadow could interfere with the precise evaluation of the sacral endplate. The rotation of the pelvis could also deform the shape of the sacral endplate to oval on the radiograph [20].

Vrtovec et al. [11] reported that, for PI measurements, 2D radiographic images showed approximately 5° overestimation compared with 3D CT images and that the manual measurements through 2D cross-section could not reflect the precise center and inclination of the 3D anatomical structure. In addition, Yamada et al. [21] analyzed the reliability of measuring spinopelvic parameters, including PI, on standing whole-spine lateral radiographs and standing lateral pelvis radiographs. They also reported that PI also tends to be larger by approximately 5° due to a large projection angle to the sacral endplate on standing whole-spine lateral radiographs compared with that on standing lateral pelvic radiographs. Chen et al. [22] also reported that, as the vertical projection point is positioned higher than the spinopelvic area on whole-spine radiographs, the femoral heads failed to align and the sacral endplate could not be sharply defined. However, on pelvic radiographs, the vertical projection point of the radiograph tube was present in the spinopelvic area, such that the femoral heads were aligned and accurate identification of the sacral endplate was possible. The optimized radiographic intensity in the pelvic area contributes to more precise visualization of the femoral heads and sacral endplate through increased signals in the pelvic area. Thus, compared with whole-spine radiographs, standing pelvis radiographs would be more effective in analyzing spinopelvic parameters, including PI.

**Fig. 7:**
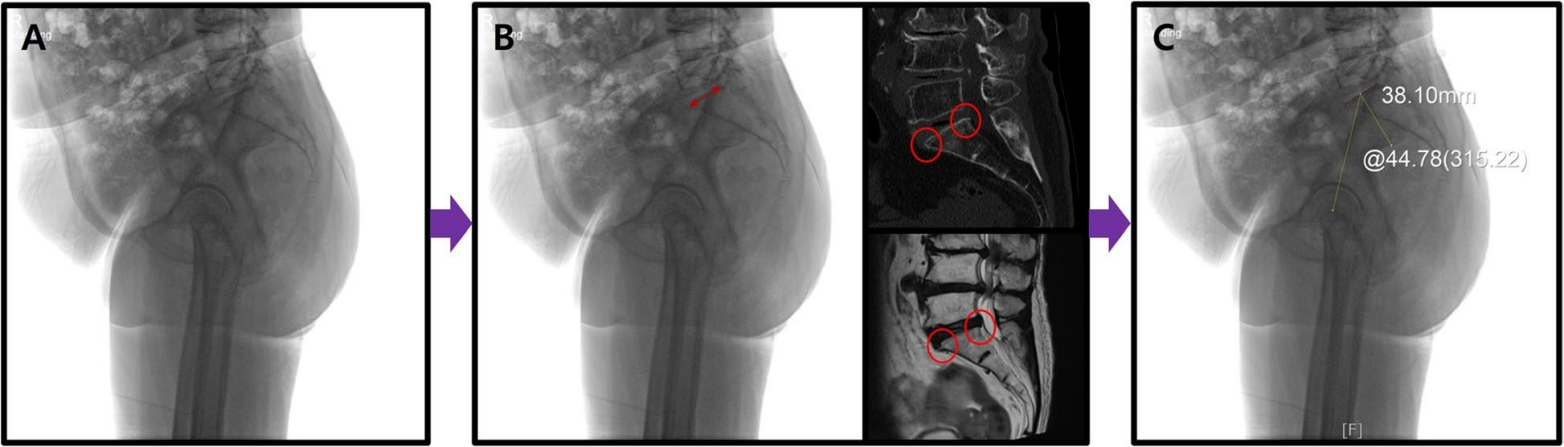
Measurement of pelvic incidence in true pelvis lateral radiographs; **A** Overlapping of both femoral heads. **B** Precise identification of sacral endplate using CT or MRI. **C** More accurate measurement of pelvic incidence

In treating patients with ASD, the measurement of the Cobb’s angle using whole-spine radiographs should be performed. However, greater emphasis is placed on standing lateral pelvic radiographs than whole-spine radiographs in evaluating spinopelvic parameters, including PI. To minimize measurement errors according to the horizontal and vertical rotation of the pelvis, the following methods are suggested for PI measurements: In producing the standing pelvic lateral radiographs, the pelvis should first be adjusted horizontally by placing the feet above a block in the case of pelvic obliquity on whole-spine radiographs. After checking the greater trochanter (GT) of the femur through palpation, the center points of the X-ray tube and cassette should be positioned approximately 3 cm above the GT at 150° to produce maximum overlapping of the two femoral heads such that they are positioned at the center of the produced images (true pelvis lateral radiograph, Fig. 7A). Even in the case of complete overlap of the two femoral heads, the first sacral endplate boundary may be unclear. In such cases, the proximal and distal boundaries of the upper endplate should be precisely identified in reference to the sagittal cut on CT or MRI of the sacral endplate, and the drawings can be made on the standing pelvic lateral radiographs (Fig. 7B). The subsequent PI measurement is anticipated to be more accurate based on the angle between the key line from the center of the sacral endplate and the line connecting the identified center of the sacral endplate and the center of the two femoral heads in maximum overlap (Fig. 7C).

### Limitations

This study has some limitations. First, as the study was conducted retrospectively, several confounding variables may exist. Second, the PI measurements were taken using only the 3D model of special mechanical programs, including AutoCAD, to prevent direct comparison with 2D radiographs. In addition, only PI was studied, unlike in previous studies [12, 20, 23, 24] on PT and SS. Futhermore, influencing factors such as the original position or orientation of each patient during the CT scan were not considered in this study. Nevertheless, more precise PI measurements using the 3D model are believed to differentiate this study from previous studies. Third, in studies using 2D radiographs, there have been few cases of pelvic rotation of 30–35° or more as in our study, and our acceptable angle was relatively large compared to that of previous studies. The results of this study were measured by artificially rotating 3D reconstruction images of patients’ CT scans, so there may be limitations and differences with 2D radiographs; therefore, a future comparative study between 3D and 2D radiographs is necessary. Fourth, with the recent advancement of novel imaging techniques, including EOS imaging (Biospace Med, France), far more accurate angle measurements have become possible. However, considering that most clinics have not yet acquired the EOS, the method based on true pelvic lateral radiographs suggested in this study is anticipated to serve as a useful guideline for spine surgeons planning surgical treatments for ASD.

## Conclusions

This study revealed that the value of the PI could differ from the actual anatomical value because of the horizontal and vertical rotation of the pelvis. Regarding whole-spine lateral radiographs, errors in PI measurements may occur due to pelvic rotation or nonvertical projection of X-rays. Therefore, PI should be measured using standing pelvic lateral radiographs instead of whole-spine radiographs to minimize measurement errors. In the standing pelvic lateral radiographs, ensuring superposition of the femoral heads at the center and obtaining the straight sacral endplate as much as possible by referring to CT or MRI scans would be a more accurate measurement method to define PI.

## Data Availability

The datasets used and/or analysed during the current study are available from the corresponding author on reasonable request.

## Abbreviations

TK: Thoracic kyphosis
LL: Lumbar lordosis
ASD: Adult spinal deformity
PI: Pelvic incidence
PT: Pelvic tilt
SS: Sacral slop
3D: 3-dimensional
2D: 2-dimensional
PACS: Picture archiving communication system
SVA: Sagittal vertical axis
ICCs: Intraclass correlation coefficient
GT: Greater trochanter
